# Modification of female and male social behaviors in estrogen receptor beta knockout mice by neonatal maternal separation

**DOI:** 10.3389/fnins.2014.00274

**Published:** 2014-09-02

**Authors:** Mumeko C. Tsuda, Naoko Yamaguchi, Mariko Nakata, Sonoko Ogawa

**Affiliations:** ^1^Laboratory of Behavioral Neuroendocrinology, University of TsukubaTsukuba, Japan; ^2^Department of Pharmacology, School of Medicine, Aichi Medical UniversityNagakute, Japan

**Keywords:** estrogen receptor β, stress, anxiety, aggression, adolescence, social anxiety, social preference, sex differences

## Abstract

Maternal separation (MS) is an animal model mimicking the effects of early life stress on the development of emotional and social behaviors. Recent studies revealed that MS stress increased social anxiety levels in female mice and reduced peri-pubertal aggression in male mice. Estrogen receptor (ER) β plays a pivotal role in the regulation of stress responses and anxiety-related and social behaviors. Behavioral studies using ERβ knockout (βERKO) mice reported increased social investigation and decreased social anxiety in βERKO females, and elevated aggression levels in βERKO males compared to wild-type (WT) mice. In the present study, using βERKO and WT mice, we examined whether ERβ contributes to MS effects on anxiety and social behaviors. βERKO and WT mice were separated from their dam daily (4 h) from postnatal day 1–14 and control groups were left undisturbed. First, MS and ERβ gene deletion individually increased anxiety-related behaviors in the open field test, but only in female mice. Anxiety levels were not further modified in βERKO female mice subjected to MS stress. Second, βERKO female mice showed higher levels of social investigation compared with WT in the social investigation test and long-term social preference test. However, MS greatly reduced social investigation duration and elevated number of stretched approaches in WT and βERKO females in the social investigation test, suggesting elevated levels of social anxiety in both genotypes. Third, peri-pubertal and adult βERKO male mice were more aggressive than WT mice as indicated by heightened aggression duration. On the other hand, MS significantly decreased aggression duration in both genotypes, but only in peri-pubertal male mice. Altogether, these results suggest that βERKO mice are sensitive to the adverse effects of MS stress on subsequent female and male social behaviors, which could then have overrode the ERβ effects on female social anxiety and male aggression.

## Introduction

Childhood exposure to an adverse environment is frequently associated with an increased risk in developing emotional and social adjustment disorders (Agid et al., 1999; Heim and Nemeroff, 2001). Maternal separation (MS) is an animal model widely used to gain an understanding in the effects of early life stress on subsequent behaviors (see reviews Sanchez et al., 2001; Millstein and Holmes, 2007; Veenema, 2012). A large number of literature report effects of MS on emotionality and anxiety-related behaviors, but the effects on social behaviors are less understood. MS procedures used in our laboratory involves the removal of pups from their mother for 3–4 h each day during the dark phase of the light/dark cycle for the first two weeks of life. With this particular MS procedure, we previously reported sex-specific effects of MS on anxiety-related and social behaviors. Specifically, MS in C57BL/6J female mice increased anxiety-related behaviors in the open field test, increased social anxiety levels toward unfamiliar opponent mice in the social investigation test, and decreased social preference toward male opponent mice in the long-term social preference test compared to non-separated mice (Tsuda and Ogawa, 2012). In C57BL/6J male mice, MS was found to greatly suppress aggression levels during the peri-pubertal period without disrupting social investigative behaviors (Tsuda et al., 2011). Taken together, our MS paradigm demonstrated that early life stress could have detrimental effects on the development of female and male social behaviors.

It is well known that estrogen can regulate a variety of behavioral and physiological functions involving reproduction (Ogawa et al., 1998, 1999, 2000; Nomura et al., 2006), cognition (Luine et al., 1998; Luine, 2008), emotionality (Fink et al., 1998), and stress responses (Critchlow et al., 1963; Bohler et al., 1990). Estrogen's various effects are mediated by two nuclear receptors, estrogen receptor α (ERα) and ERβ (Green et al., 1986; Kuiper et al., 1996). Areas such as the bed nucleus of the stria terminalis, amygdala, medial preoptic nucleus, and locus coeruleus express both forms of ER, however the supraoptic nucleus and paraventricular nucleus of the hypothalamus (PVN) exclusively contains ERβ and nearly no ERα (Shughrue et al., 1996, 1997; Mitra et al., 2003). The expression of ERβ in the above-mentioned brain regions suggests for a potential involvement in the regulation of anxiety-related and social behaviors, as well stress responses.

Numerous studies have provided evidence for the potential role of ERβ in the regulation of anxiety levels as well as social behaviors (Handa et al., 2012). Studies using ERβ null mice (βERKO) have reported increased anxiety-related behaviors in βERKO female mice compared to their wild-type counterparts in the open field, elevated plus maze, and light-dark transition tests (Krezel et al., 2001; Imwalle et al., 2005; Tomihara et al., 2009). These results are indicative that ERβ has anxiolytic effects in nonsocial tests. On the other hand, reduced anxiety-related behaviors are observed in βERKO female mice in social conditions. In social recognition tests, βERKO female mice persistently showed high levels of social investigation and reduced number of stretched approaches (an index for anxiety levels) to a repeatedly presented conspecific (Choleris et al., 2003), suggesting reduced social anxiety. Therefore, depending on the context of the test, i.e., nonsocial vs. social, there are differential effects of ERβ on anxiety-related behaviors. Besides the involvement with anxiety behaviors, ERβ has also been shown to be a key player in the regulation of aggressive behaviors. For example, βERKO male mice exhibit increased levels of aggression, depending on their social experience and age (Ogawa et al., 1999; Nomura et al., 2002a, 2006), suggesting that ERβ may play an inhibitory role in the regulation of male aggressive behavior.

During the neonatal period, various factors might contribute to the effects of MS on subsequent behavioral and neuroendocrine functions. Genetic factors such as ERβ may be a possible candidate because of its known role in regulating stress responses, anxiety-related behaviors, and social behaviors. High levels of ERβ are detected in the PVN between postnatal days 1–9 (Zhang et al., 2004; Zuloaga et al., 2014), which coincides with the postnatal development of the hypothalamic-pituitary-adrenal (HPA) axis, the major regulatory system that controls reactions to stress (Schmidt et al., 2003). Furthermore, MS stress causes lasting alternations in HPA activity, in which MS rats and mice display augmented HPA function under basal and stressful conditions (Wigger and Neumann, 1999; Kalinichev et al., 2002; Parfitt et al., 2004). Therefore, it is possible that ERβ is involved in MS effects on the development of the HPA axis and any subsequent behaviors. To assess whether MS stress differentially affects mice lacking functional ERβ, we investigated the effects of MS on female and male anxiety-related behaviors in the open field test, female social behaviors in the social investigation and social preference tests, and male peri-pubertal and adult aggression of βERKO mice subjected to neonatal MS stress.

## Materials and methods

### Animals

Adult female heterozygous (HZ) mice were mated with either βERKO or wild-type (WT) male mice. This specific mating scheme was necessary to obtain enough number of WT and βERKO pups in each treatment group. βERKO male mice were viable mating partners because they display normal male sexual behavior similar to WT mice (Ogawa et al., 1999). βERKO, WT, and HZ mice used for mating were obtained from βERKO breeding colonies maintained at the University of Tsukuba. Original HZ breeding pairs were obtained from the National Institute of Environmental Health Sciences (Research Triangle Park, NC, USA) and completely backcrossed to C57BL/6J mice (Krege et al., 1998). During the last week of gestation, pregnant HZ females were individually housed in plastic cages (29 × 19 × 12 cm) with nesting material and monitored daily for parturition. The day of parturition was defined as postnatal day (PND) 0. Stimuli mice used for behavioral testing were either C57BL/6J or ICR/Jcl from CLEA (Tokyo, Japan). All mice were maintained on a 12:12 light/dark cycle (lights off at 1200) and at a constant temperature (23 ± 2°C) throughout the study. Food and water were provided *ad libitum*. All procedures in this study were conducted with approval from the Animal Care and Use Committee and the Recombinant DNA Use Committee at the University of Tsukuba and strictly followed the National Institutes of Health guidelines.

### Maternal separation procedures

MS procedures were followed as previously described in detail in Tsuda and Ogawa (2012). Briefly, on PND 1, each litter was culled to six pups (2–4 females in each litter) and assigned to either a control or MS group. From PND 1 to 14, MS pups were removed together into a small container placed on a warmer maintained a constant temperature of 36°C and separated from their dam for 4 h each day between 1500 and 1900. Control pups remained with their dam. On PND 21, all pups were ear punched, weaned and group-housed with littermates of the same sex. Tail samples were collected at this time for genotyping (Krege et al., 1998). Only βERKO and WT mice obtained from the respective HZ × βERKO and HZ × WT mating schemes were used for behavioral testing.

### Experimental groups

At 12 weeks of age, female offspring were ovariectomized (OVX) under general anesthesia with isoflurane inhalation (Dainippon Sumitomo Pharma, Japan) and single-housed at this time. At 13 weeks of age, anxiety-related behaviors in WT (control = 7; *MS* = 7) and βERKO (control = 7; *MS* = 11) female mice were measured in the open-field test (OFT). Following OFT, female mice were tested for social investigative behaviors toward an unfamiliar female opponent in the social investigation test (SIT) and social preference for female and male stimuli in a long-term social preference test (SPT) at 14–15 weeks of age.

Male mice were single-housed one week before testing and left as gonadally intact. At 13 weeks of age, WT (control = 9; *MS* = 6) and βERKO (control = 5; *MS* = 8) male mice were tested for anxiety-related behaviors in OFT. Following OFT, male mice were examined for adult male aggression at 14 weeks of age. A separate cohort of male mice was used to investigate peri-pubertal male aggression in WT (control = 10; *MS* = 9) and βERKO (control = 8; *MS* = 9) mice at 5 and 6 weeks of age. All behavior tests, unless otherwise noted, were tested during the dark phase (1400–1800, at least 2 h after lights off).

### Open-field test (OFT)

The open-field arena (60 × 60 × 30 cm) was illuminated to 5 lux and the floor was hypothetically divided into 25 equal square sections, 9 inner sections (center area) and 16 outer sections (peripheral area). Mice were placed in the corner and activity was monitored for 10 min on a Macintosh computer using Image OFC 2.03 (O'Hara & Co., Ltd., Tokyo, Japan), modified software based on the public domain NIH Image program (developed at the U.S. National Institutes of Health and available on the internet at http://rsb.info.nih.gov/nih-image/). Total moving distance was analyzed as a measure of activity and time spent in the center area was used as an index of anxiety.

### Social investigation test (SIT)

SIT apparatus (SOSI Type1, O'Hara & Co., Ltd., Tokyo, Japan) and methods are described in detail in Tsuda and Ogawa (2012). Social investigative behaviors of female mice were assessed against a cylinder containing an unfamiliar OVX female C57BL/6J mouse placed in the center of their home cage for 15 min. Cylinders used to introduce stimulus mice were made of clear Plexiglas and were perforated near the bottom (Mouse Cylinder SIOT1, O'Hara & Co., Ltd., Tokyo, Japan). All tests were video recorded and scored off-line using a digital event recorder program (Recordia 1.0b, O'Hara & Co., Ltd., Tokyo, Japan). All mice were analyzed for measurements of social investigation duration and number of stretched approaches. Detailed description of behaviors are presented in Tsuda and Ogawa (2012). One control WT female was excluded from analysis due to no activity during testing.

### Long-term social preference test (SPT)

Female mice were tested in a long-term SPT (AMAZENG TYPE1, O'Hara & Co., Ltd., Tokyo, Japan) as previously described in detail by our laboratory (Tsuda and Ogawa, 2012). The apparatus consisted of a large plastic cage (test mice) connected to two smaller cages (stimuli mice) by a tunnel. Wire mesh between tunnel and small cage prevented physical contact. Social preference between an OVX ICR/Jcl female and a gonadally intact ICR/Jcl male mouse was continuously measured for 5 days. The time experimental mice spent in each tunnel were recorded on a Windows computer using the Time BAP software (O'Hara & Co., Ltd., Tokyo, Japan). Cumulative duration spent in each tunnel during the 12 h dark phase was analyzed and averaged for the testing period. One MS WT and one control βERKO female mouse displayed a strong preference (>85%) for the same smaller cage during both baseline and testing periods and were excluded from analysis. Throughout SPT, all mice were provided with food and water *ad libitum*.

### Aggressive behavior test

Male aggression was assessed in a resident-intruder paradigm for 2 consecutive days at 5 and 6 weeks of age (peri-pubertal mice) or 3 consecutive days at 14 weeks of age (adult mice) under red lighting. Resident mice were tested in their home-cage against a weight-matched, group-housed, gonadally intact, olfactory bulbectomized (OBX) C57BL/6J intruder male mouse for 15 min. Resident mice encountered a different intruder mouse in each aggression test. OBX intruder males rarely display aggression but are capable of eliciting aggression from resident mice. Therefore, OBX stimuli mice eliminate possible confounding effects of social defeat experience. All tests were videotaped and scored for the number of aggressive bouts, cumulative duration of aggressive bouts, and latency to the first aggressive bout using the Recordia 1.0 b program. Data for each week were averaged for each mouse. An aggressive bout was defined as a series of behavioral interactions consisting of at least one of the following: chasing, boxing, tail rattling, wrestling, biting, and offensive lateral attack. If more than 3 s elapsed between aggressive bouts, they were scored as two separate bouts.

### Statistics

OFT, SIT, and adult male aggression data were analyzed by a Two-Way ANOVA for main effects of treatment, genotype, and their interactions within each sex. Long-term SPT data were analyzed with either a Two-Way ANOVA for treatment and genotype differences in the combined time spent with both stimuli mice or a paired t-test to compare the differences in time spent investigating between paired stimulus mice. Peri-pubertal male aggression data were analyzed by a Three-Way ANOVA for repeated measurements for the main effects of treatment, genotype, age, and their interactions. Significant ANOVA interactions were followed by Bonferroni *post-hoc* tests and significant main effects were analyzed as a separate ANOVA for each main effect. Significant differences were considered when *p* < 0.05. All data were analyzed using SPSS 14.0J (SPSS, Chicago, IL) statistical package. All data are presented as mean ± standard error of the mean (SEM).

## Results

### Anxiety-related behaviors

#### Females

**Figure 1:**
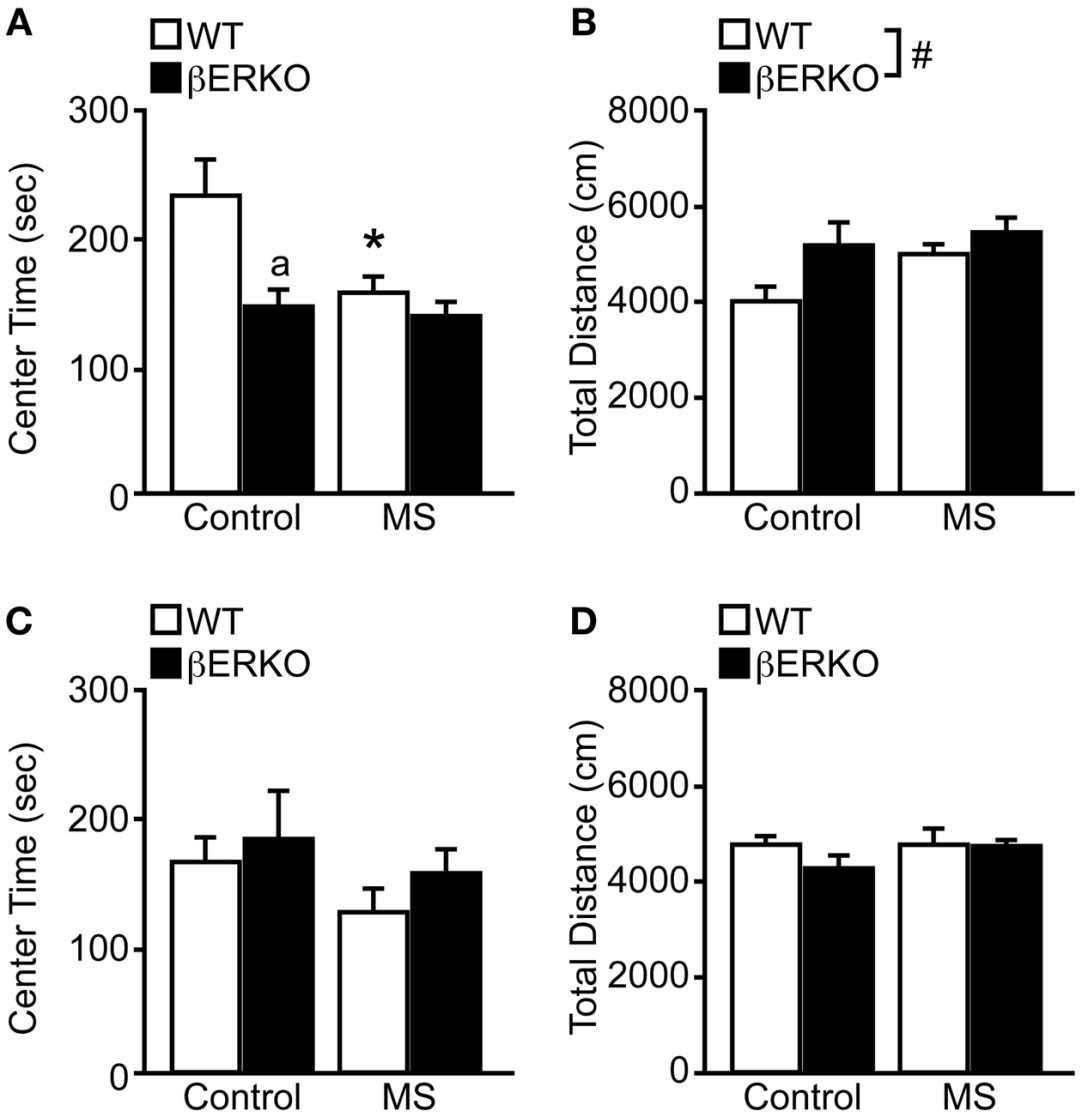
**MS effects on anxiety-related behaviors measured in the OFT in female and male WT and βERKO mice**. **(A,B)** Female and **(C,D)** male WT and βERKO mice. **(A,C)** The total time spent in the center area and **(B,D)** total moving distance in the entire arena measured during OFT. All data are presented as mean ± s.e.m. ^*^p < 0.05 vs. control of same genotype; ^a^*p* < 0.05 vs. WT of same treatment group; ^#^*p* < 0.05.

In OFT, there were significant main effects of MS and genotype on the time spent in center area ([treatment: *F*_(1, 28)_ = 5.06, *p* < 0.05; genotype: *F*_(1, 28)_ = 8.24, *p* < 0.01], Figure 1A) and also a marginally significant interaction of treatment and genotype [*F*_(1, 28)_ = 3.57, *p* = 0.07]. *Post-hoc* analysis revealed that MS reduced the time spent in the center area only in WT mice compared to the control group (*p* < 0.05), and the center time of βERKO only differed from WT in the control group (*p* < 0.05). These results indicate that MS stress and ERβ deficiency may independently increase anxiety-related behaviors in female mice.

On the other hand, only genotype differences were found in the total moving distance ([*F*_(1, 28)_ = 4.69, *p* < 0.05], Figure 1B), in which βERKO mice were more active than WT regardless of treatment. Although βERKO female mice moved more during OFT, it is notable that in the control group, βERKO mice also spent less time in the center area. This may indicate an abnormal response of βERKO female mice to a novel environment in the OFT.

#### Males

In both behavioral measurements of time spent in the center area and total moving distance, no significant effects of treatment or genotype were found in male mice (Figures 1C,D). Therefore, neither MS nor ERβ gene deletion affected anxiety levels measured in OFT in male mice.

### Female social investigative behaviors

**Figure 2:**
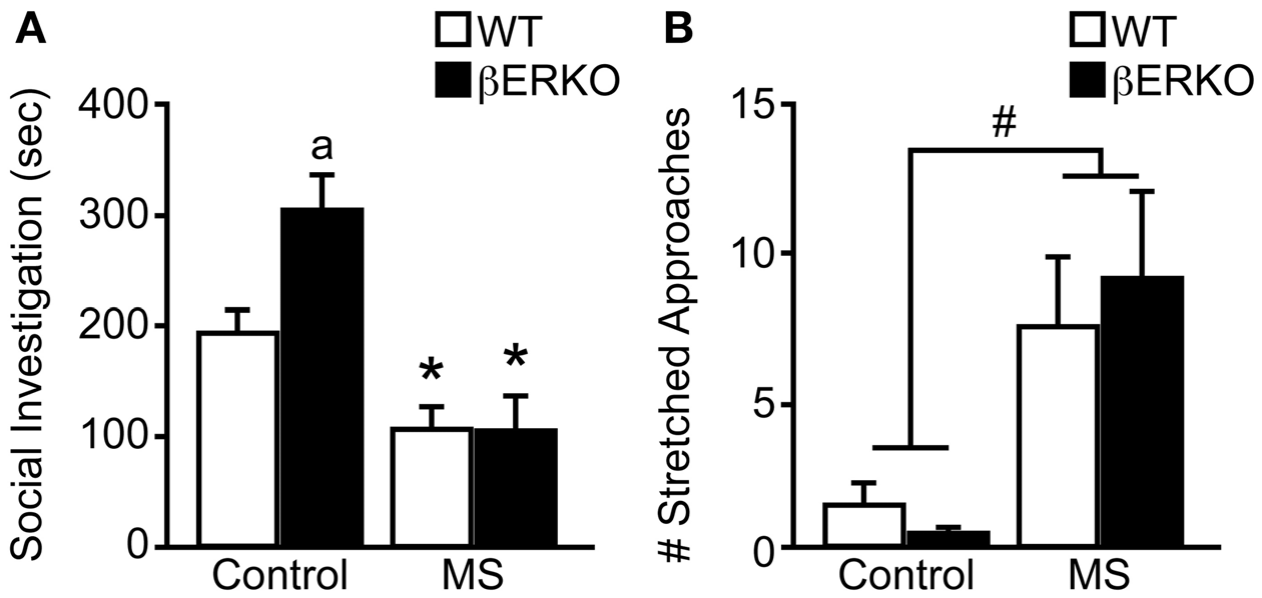
**Genotype and MS effects on social investigative behaviors during SIT. (A)** Cumulative social investigation duration and **(B)** number of stretched approaches toward an unfamiliar female opponent mouse in SIT. All data are presented as mean ± s.e.m. ^*^p < 0.05 vs. control of same genotype; ^a^*p* < 0.05 vs. WT of same treatment group; ^#^*p* < 0.05.

Similar to our previously reported findings (Tsuda and Ogawa, 2012), MS increased social anxiety levels toward unfamiliar female stimuli mice in SIT. Specifically, MS greatly reduced social investigation duration ([treatment: *F*_(1, 28)_ = 19.77, *p* < 0.001], Figure 2A) and significantly increased the number of stretched approaches ([treatment: *F*_(1, 28)_ = 8.62, *p* < 0.01], Figure 2B). Although no significant effect of genotype was found in either behavioral parameter, there was a marginally significant interaction of treatment and genotype in social investigation duration [*F*_(1, 28)_ = 3.09, *p* < 0.08]. *Post-hoc* analysis showed that control βERKO female mice spent more time sniffing the stimuli-containing cylinder compared to control WT mice (*p* < 0.05). However, MS greatly diminished social investigation duration in βERKO compared to control group (*p* < 0.05) and eliminated any genotype differences. Moreover, MS-induced reduction in social investigation duration was much greater in βERKO (61.88%) mice than WT mice (45.61%), suggesting that MS effects on social investigative behaviors were more apparent and possibly more adverse in mice that lack ERβ gene function.

### Female social preference

**Figure 3:**
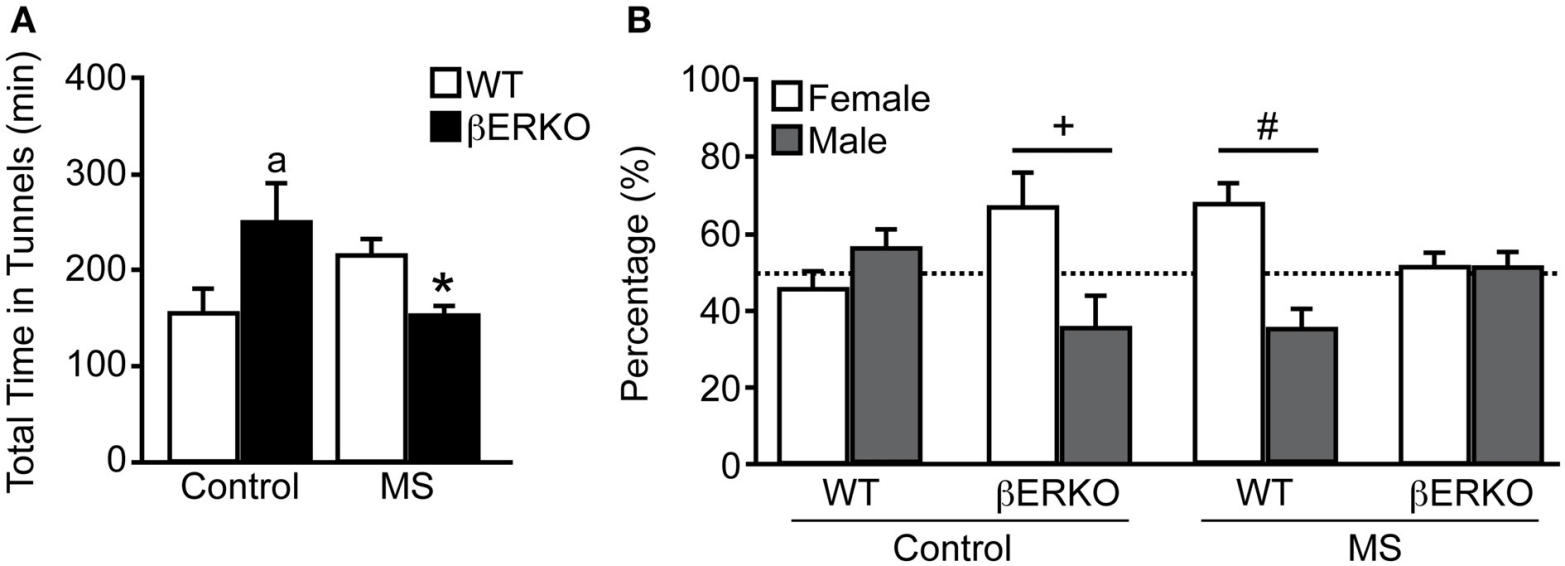
**Effects of MS and genotype on social preference during long-term SPT. (A)** Total time spent in both tunnels and **(B)** percent of time spent in each tunnel connected to unfamiliar female and male stimuli cages. All data are presented as mean ± s.e.m. ^*^p < 0.05 vs. control of same genotype; ^a^*p* < 0.05 vs. WT of same treatment group; ^#^*p* < 0.05; ^+^*p* = 0.06.

There was a significant interaction of treatment and genotype on the total time females spent in the two tunnels connected to female and male stimuli mice in long-term SPT ([treatment: n.s.; genotype: n.s.; treatment × genotype: *F*_(1, 28)_ = 9.05, *p* < 0.01]; Figure 3A). Control βERKO females spent more time in the tunnels compared to control WT (*p* < 0.05). In WT female mice, MS did not affect the total time spent in the tunnels whereas MS greatly reduced it in βERKO mice (*p* < 0.05). Altogether, lack of ERβ increased social interest toward unfamiliar opponents in female mice, but this phenotype was suppressed (or attenuated) when βERKO females experienced neonatal MS stress.

While control WT females displayed no preference for either stimuli sex, control βERKO [*t*_(5)_ = 2.54, *p* = 0.06] mice displayed a preference for female over male stimuli (Figure 3B). In MS groups, WT female mice showed a preference for female over male [*t*_(6)_ = 2.88, *p* < 0.05], but βERKO mice failed to show any preference for either stimuli mice. Greatly reduced total time spent in tunnels found in βERKO mice that underwent MS stress (Figure 3A) was actually due to the decreased time spent with the female opponent mouse.

### Peri-pubertal male aggression

**Figure 4:**
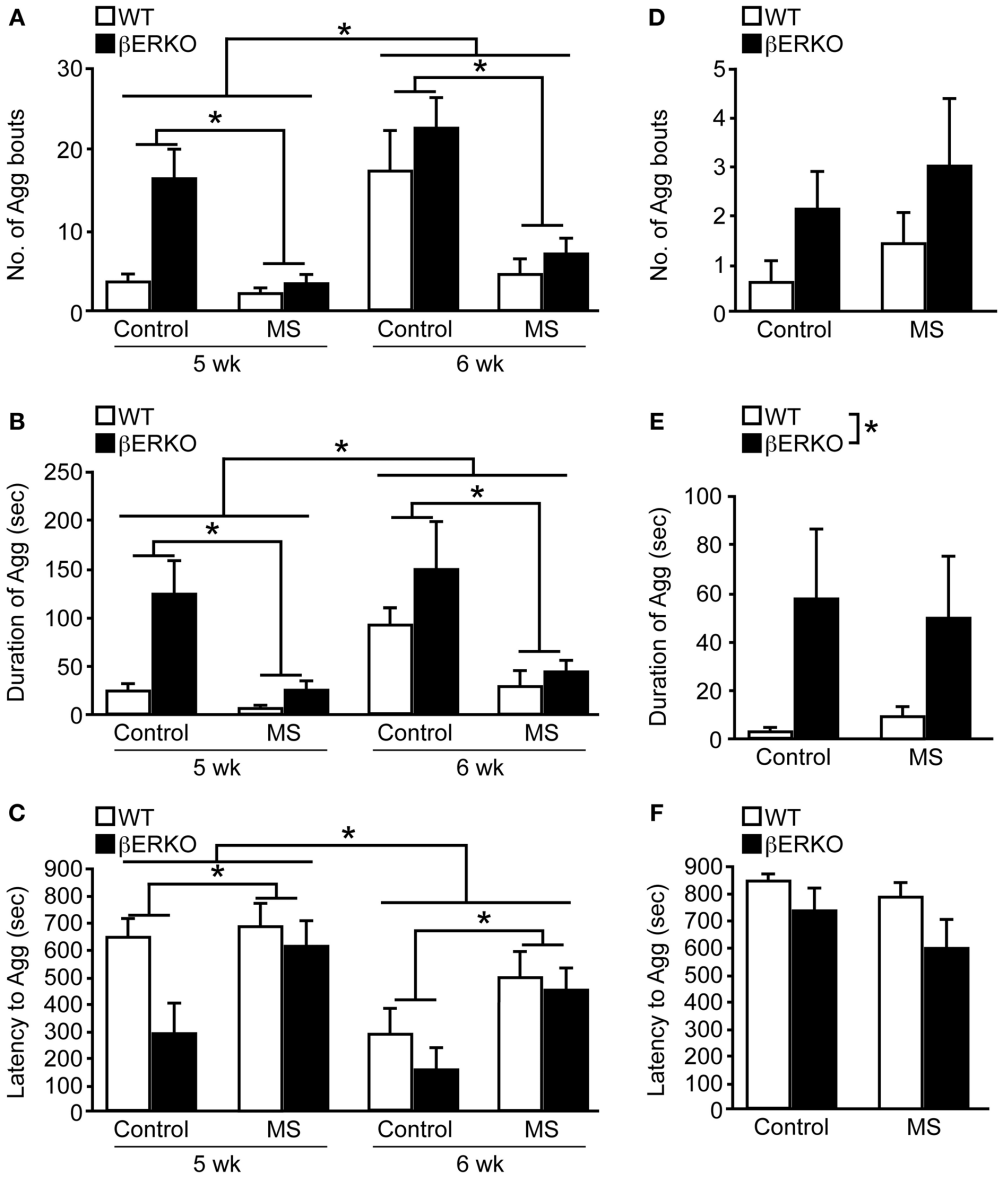
**MS effects on peri-pubertal and adult male aggression. (A–C)** Peri-pubertal and **(D–F)** adult WT and βERKO male mice. **(A,D)** Number of aggressive bouts, **(B,E)** cumulative duration of aggression, and **(C,F)** latency to the first aggressive bout. All data are presented as mean ± s.e.m. ^*^p < 0.05.

Peri-pubertal male aggressive behaviors were greatly suppressed by MS stress in both βERKO and WT mice at 5 and 6 weeks of age. There was a significant main effect of MS and age on the number of aggressive bouts ([treatment: *F*_(1, 29)_ = 11.03, *p* < 0.01; age: *F*_(1, 29)_ = 23.95, *p* < 0.0001]; Figure 4A), cumulative duration of aggression ([treatment: *F*_(1, 29)_ = 10.37, *p* < 0.01; age: *F*_(1, 29)_ = 10.82, *p* < 0.01]; Figure 4B), and latency to the first aggressive bout ([treatment: *F*_(1, 29)_ = 3.14, *p* = 0.09; age: *F*_(1, 29)_ = 17.45, *p* < 0.01]; Figure 4C), in which aggression levels were greater at 6 weeks of age compared to 5 weeks. However, no effect of genotype or interactions was found in all three behavioral measurements.

Further detailed analysis within each week revealed that consistent with previously reported findings (Nomura et al., 2002a), control βERKO males were more aggressive compared to their WT counterparts at 5 weeks of age, as indicated by higher numbers of aggressive bouts (*p* < 0.05), increased cumulative duration of aggression (*p* < 0.05), shorter latency to the first aggressive bout (*p* < 0.05). There were no longer genotype differences in control mice at 6 weeks of age, possibly due to higher aggression levels in WT male mice at this age. During each week of testing, MS greatly reduced the levels of aggression in both genotypes. Particularly, MS βERKO males showed significantly lower number of aggressive bouts (5 weeks, *p* < 0.01; 6 weeks, *p* < 0.05) and cumulative duration of aggression (5 weeks, *p* < 0.01; 6 weeks, *p* < 0.05) compared to control males at both 5 and 6 weeks of age. In WT mice, MS males also showed significantly lower number of aggressive bouts compared to control males at 6 weeks (*p* < 0.05), but no MS effect was detected at 5 weeks possibly due to low levels of aggression in the control group.

### Adult male aggression

In contrast to peri-pubertal male aggression, there were no effects of MS found in all three behavioral measurements (Figures 4D–F) of adult male aggression. On the other hand, regardless of treatment, βERKO males were found to display significantly higher levels of aggression than WT mice as measured by cumulative duration of aggression ([*F*_(1, 24)_ = 5.61, *p* < 0.05], Figure 4E). Latency to the first aggressive bout was also marginally shorter in βERKO compared to WT mice ([*F*_(1, 24)_ = 3.34, *p* = 0.08], Figure 4F).

## Discussion

The present study provides two major findings. First, we confirmed that MS stress and ERβ gene deletion could individually modify female anxiety-related and social behaviors and male aggression. Specifically, MS increased female anxiety levels in OFT and social anxiety levels in SIT, and reduced peri-pubertal male aggression. On the other hand, ERβ gene knockout elevated anxiety in OFT and increased investigative behaviors in SIT and SPT of females and heightened adult and peri-pubertal male aggression. Second, social behavior alterations found during SIT and SPT in βERKO females and aggression tests in βERKO males were overruled by MS stress, but not anxiety-related behaviors in OFT (Table 1). These results suggest that social behaviors of βERKO mice are vulnerable to MS and can be modified by the adverse effects of early life stress.

**Table 1:** **Summary table describing effects of ERβ gene deletion, MS, and interaction of ERβ gene deletion and MS on anxiety-related and social behaviors in female and male mice**.

	**ER β gene deletion**	**MS**	**ERβ gene deletion × MS**
**FEMALES**			
Open-field test	↑ Anxiety	↑ Anxiety	↑ Anxiety
Social investigation test	↑ Social investigation	↓ Social investigation	↓ Social investigation
	↓ Social anxiety	↑ Social anxiety	↑ Social anxiety
Social preference test	↑ Female preference	↑ Female preference	No preference
**MALES**			
Open-field test	↔ Anxiety	↔ Anxiety	↔ Anxiety
Peri-pubertal aggression	↑ Aggression	↓ Aggression	↓ Aggression
Adult aggression	↑ Aggression	↔ Aggression	↑ Aggression

*Arrows denote differences relative to control WT mice*.

### Sex-dependent effects of MS and genotype on anxiety-related behaviors

Anxiety-related behaviors measured during OFT in female mice demonstrated that MS increased anxiety levels in a novel environment, which supports our previously published findings in OVX C57BL/6J female mice (Tsuda and Ogawa, 2012). Furthermore, control βERKO females also displayed enhanced anxiety levels compared to WT in OFT. These findings are consistent with previous studies that reported elevated anxiety-related behaviors in gonadally intact and OVX βERKO female mice in the elevated plus maze, OFT (Krezel et al., 2001; Imwalle et al., 2005) and light-dark transition tests (Tomihara et al., 2009), which suggests an ERβ involvement in anxiolysis in females. Given that both MS stress and ERβ gene deletion increased anxiety-related behaviors in OFT, βERKO females subjected to MS did not show amelioration or augmentation in anxiety levels. It may be possible that there is a threshold for anxiety beyond which no further increase can be measured in OFT. Therefore, it is possible that lack of ERβ during neonatal MS stress may have further contributed to already heightened levels of anxiety, but this effect was not measurable in OFT. Whether anxiety levels of βERKO females are indeed more susceptible to MS need to be further investigated using light-dark box transition, elevated zero maze, and/or elevated plus maze tests, which are widely used to examine anxiety levels in mice.

Few studies to date address sex differences of MS effects. As gonadally intact, studies report a stronger effect of MS in males rather than females (Wigger and Neumann, 1999; Kalinichev et al., 2002; Kundakovic et al., 2013) or no sex differences in anxiety levels measured in OFT and elevated plus maze (Rhees et al., 2001; Millstein and Holmes, 2007; Veenema et al., 2007). However, Romeo et al., found that MS increased anxiety in males, but decreased anxiety in diestrus (low estrogen) females (Romeo et al., 2003), suggesting endogenous estrogen levels may influence MS effects in females. In our study, females were tested as OVX to eliminate confounding effects of endogenous estrogen. It was never the intention to directly compare female and male littermates since hormonal conditions differ, but rather to examine whether MS affected female and male mice differently. Indeed, our findings demonstrated that MS effects on anxiety levels were stronger in females than males in OFT. The differential effects of MS in females may be due to differences in estrogen levels at the time of testing, i.e., no estrogen increases anxiety (OVX), low levels of estrogen (diestrus) decreases anxiety, and high levels of estrogen (estrus) has no effect in OFT. Interestingly, ERβ involvement in regulating anxiety levels may also depend on estrogen levels. High doses of estrogen were anxiogenic in both WT and βERKO females, but low doses of estrogen were anxiolytic only in WT and not βERKO females (Tomihara et al., 2009), which suggests that low doses of estrogen may decrease anxiety through ERβ activation. Therefore, MS effects on female anxiety may be associated or dependent on estrogen levels at the time of testing and may involve ERβ's estrogenic action.

### Effects of MS and genotype on female social behaviors

In SIT, control βERKO females displayed a substantial increase in social investigation levels toward an unfamiliar female stimuli mouse, suggesting heightened social reactivity in βERKO mice. Previous studies have described βERKO females to persistently display high levels of social investigation and reduced counts of stretched approaches toward familiar stimuli mice in social recognition and binary choice tests, suggesting that loss of ERβ function induces a hyper-reactive and low social anxiety phenotype in female mice (Choleris et al., 2003, 2006). However, this behavioral phenotype of βERKO females was eliminated with neonatal MS stress experience. MS reduced social investigation duration and increased number of stretched approaches toward an unfamiliar stimulus mouse in SIT in WT, supporting our previously published observations of elevated social anxiety in C57BL/6J female mice (Tsuda and Ogawa, 2012). Moreover, these same behavioral alterations induced by MS stress were found in MS βERKO female mice, suggesting MS overturned the socially hyper-reactive phenotype of control βERKO mice.

Both control βERKO and MS WT mice significantly preferred a female mouse to a male mouse in SPT, whereas control WT exhibited no social preference. However, MS βERKO females displayed no preference for either mouse and also spent less time in the tunnels connected to the stimuli cages. Enhanced female preference found in control βERKO may be correlated with high social reactivity to a female opponent observed in SIT. We previously reported that the distinct preference for female stimuli to male stimuli or an empty cage during SPT in C57BL/6J MS female mice might have been due to increased social anxiety toward male opponents (Tsuda and Ogawa, 2012). In Tsuda and Ogawa (2012), MS females displayed more social anxiety-like behaviors to male stimuli in SIT and showed no preference between a male mouse and an empty cage in long-term SPT. It is possible that the MS-induced increase in social anxiety may have been more prominent in βERKO females and contributed to the loss of social preference to both male and female stimuli in SPT. Future studies need to assess if MS effects on social anxiety levels differ between female and male opponents in βERKO mice and also evaluate if increased social anxiety in MS βERKO females contribute to a social phobia phenotype.

Heightened social anxiety levels in MS female mice were associated with increased neuronal activity (FosB expression) in the PVN, medial amygdala, and central amygdala following exposure to an unfamiliar social stimuli, while no baseline differences were found between treatment groups (Tsuda and Ogawa, 2012). Increased FosB induction in these regions was also found to be dependent on stimulus gender. Higher number of FosB cells was induced in the PVN with male stimuli exposure and in the medial amygdala with female stimuli exposure. These particular brains regions express ERβ, oxytocin, vasopressin, and corticotropin-releasing hormone, which are involved in the regulation of stress responses and social behavior (Shughrue et al., 1997; Ferguson et al., 2001, 2002; Mitra et al., 2003; Bielsky et al., 2004; Merchenthaler et al., 2004; Neumann, 2008; Milner et al., 2010). Elevated FosB induction in these brain regions possibly indicates a functional alteration of these neuroendocrine correlates in MS females. Furthermore, ERβ is co-localized and regulates oxytocin, vasopressin, and corticotropin-releasing hormone levels in these brain regions (Nomura et al., 2002b; Miller et al., 2004; Murakami et al., 2011). Thus, it is possible that MS induced alterations in female social behaviors are associated with changes in ERβ, oxytocin, vasopressin, corticotropin-releasing hormone in the amygdala and PVN and these neuroendocrine modifications are dependent on the regulatory role of ERβ.

### Effects of MS and genotype on peri-pubertal and adult male aggression

Consistent with our previous study (Tsuda et al., 2011), MS disrupted the development of peri-pubertal male aggression by suppressing levels of aggressive behavior in WT mice at 5 and 6 weeks of age. However, MS did not affect male aggression assessed in adulthood. This result is surprising since the two other studies that examined MS effects on adult male aggression in mice reported decreased male aggressive behaviors (Veenema et al., 2007; Hohmann et al., 2013). Differences in results may have been due to differences in procedures of MS and aggressive behavior testing. In particular, Veenema et al. (2007) and Hohmann et al. (2013) conducted MS during the light phase of the circadian cycle, but our MS was performed during the dark phase. Therefore, the data obtained in the present study make it difficult to directly compare results, but differences in the effects of MS between these studies demonstrates an intriguing effect of circadian phase as a potential variable to determine MS effects on adult male aggression.

Nomura et al. (2002a) reported higher levels of aggression in βERKO male mice compared to WT in pubertal (5 weeks of age) and young adult (12 weeks of age) mice, but not in adult (19 weeks of age) mice. Similarly, the present study demonstrated increased aggression levels in pubertal (4–5 weeks of age) and young adult (14 weeks of age) control βERKO male mice compared to their WT counterparts. Moreover, lack of ERβ may have advanced the pubertal onset of aggression in male mice. Control βERKO males displayed high levels of aggression already at 5 weeks of age, whereas WT males began to exhibit aggression at 6 weeks of age. Despite the strong aggressive behavior phenotype of βERKO males, neonatal MS stress remarkably suppressed aggression levels in βERKO mice, but only in peri-pubertal males and not young adult males. These results suggest that neonatal MS stress can suppress or attenuate the aggressive phenotype of βERKO male mice, at least during the pubertal period.

ERβ activation via estrogen increases oxytocin, but decreases vasopressin gene expression in the PVN of male mice (Nomura et al., 2002b). Oxytocin and vasopressin are reported to inhibit and facilitate, respectively, male aggression (Ferris, 2005). Furthermore, elevated aggression levels in pubertal βERKO male mice were associated with increased serum testosterone levels (Nomura et al., 2002a) and testosterone levels are known to be positively correlated with aggression in male rodents (Burge and Edwards, 1971). This regulatory role of ERβ on oxytocin and vasopressin expression in the PVN and plasma testosterone levels provides a potential mechanism of elevated aggression levels in βERKO male mice. On the contrary, we recently reported that lower levels of aggression in MS peri-pubertal males were associated with increased and decreased oxytocin and vasopressin positive cells in the PVN, respectively, and reduced serum testosterone levels in 4- to 6- weeks old male mice (Tsuda et al., 2011). MS induced changes in oxytocin, vasopressin, and testosterone may have outweighed or suppressed the phenotype of these hormones found in control βERKO males, resulting in less aggressive βERKO mice. To gain a stronger understanding of the possible neuroendocrine mechanisms in which MS may override ERβ effects on pubertal male aggression, MS effects on ERβ activity during the postnatal period need to be determined. Additionally, whether βERKO males subjected to MS stress display alterations in oxytocin, vasopressin, and serum testosterone levels similar to that of MS WT mice need to be evaluated to comprehend the reduced levels of aggression in pubertal male mice.

## Conclusions

The present findings demonstrated that ERβ gene deletion and MS could individually modify anxiety and social behaviors in mice. However, behavioral phenotypes of βERKO mice were overturned by MS stress in exception to nonsocial anxiety. βERKO mice could be more sensitive to the stressful effects of MS because ERβ also functions to attenuate HPA reactivity to stress (Lund et al., 2005, 2006). The lack of ERβ's inhibitory function during MS may have increased vulnerability to the stressful effects of MS on female social behaviors and male aggression, but there are still other possible mechanisms that need to be investigated. The postnatal period is a critical time of brain development and factors such as genetics and environmental conditions are significantly influential. Findings in the present study demonstrate that there is a potential role for ERβ in MS effects on certain social behaviors and contribute to our understanding of MS effects on female and male social behaviors. The combination of ERβ gene deletion and neonatal MS stress possibly involves a variety of changes in neuroendocrine systems modulating female and male social behaviors in a complex manner that further investigation is needed to fully understand the effects of early life stress on social behaviors.

## Author contributions

Mumeko C. Tsuda and Sonoko Ogawa conceived and designed the experiments; Mumeko C. Tsuda performed the experiments; Mumeko C. Tsuda, Naoko Yamaguchi, and Mariko Nakata analyzed the data, Mumeko C. Tsuda and Sonoko Ogawa contributed materials, reagents, and mice, and Mumeko C. Tsuda and Sonoko Ogawa wrote the manuscript.

### Conflict of interest statement

The authors declare that the research was conducted in the absence of any commercial or financial relationships that could be construed as a potential conflict of interest.
